# Lightning-induced keraunographic injury with otologic involvement in skin of color

**DOI:** 10.1016/j.jdcr.2026.06.056

**Published:** 2026-07-02

**Authors:** Marco A. Montoya-Flores, Kyle Mueller, Andrew S. Williams, John Trinidad

**Affiliations:** a Harvard Medical School, Boston, Massachusetts; b Universidad Peruana Cayetano Heredia, Facultad de Medicina, Lima, Peru; c Department of Dermatology, Massachusetts General Hospital, Boston, Massachusetts

**Keywords:** Fitzpatrick phototype, keraunographic injury, Lichtenberg figures, lightning injury, otologic injury, postinflammatory dyspigmentation, resource-limited settings, sensorineural hearing loss, skin of color

## Case description

A 60-year-old man with darker skin presented to a rural clinic in the Peruvian Andes 48 hours after surviving a lightning strike while walking during a thunderstorm. He reported pain but remained ambulatory, with no loss of consciousness, cardiac symptoms, altered mental status, or weakness on evaluation. Cutaneous examination revealed linear and curvilinear erythematous-violaceous lesions extending from the right postauricular and supraclavicular region across the anterior chest to the left lower abdomen (Fig 1, *A* and *B*), with focal superficial erosions near the left inguinal region (Fig 2). Dried blood near the right external auditory canal suggested otologic injury (Fig 3). No classic Lichtenberg figures were identified on examination. The patient was treated with oral analgesics and topical silver sulfadiazine. At follow-up more than 3 years later, lesions had resolved without scarring, with residual postinflammatory dyspigmentation along the original keraunographic distribution, including the anterior chest and left lateral thigh (Fig 4, *A*-*C*). Weber testing lateralized to the left, and Rinne testing was positive bilaterally; findings clinically consistent with right-sided sensorineural hearing loss. Audiometry was unavailable. The patient remained independent but reported persistent right-sided deafness.

**Fig 1:**
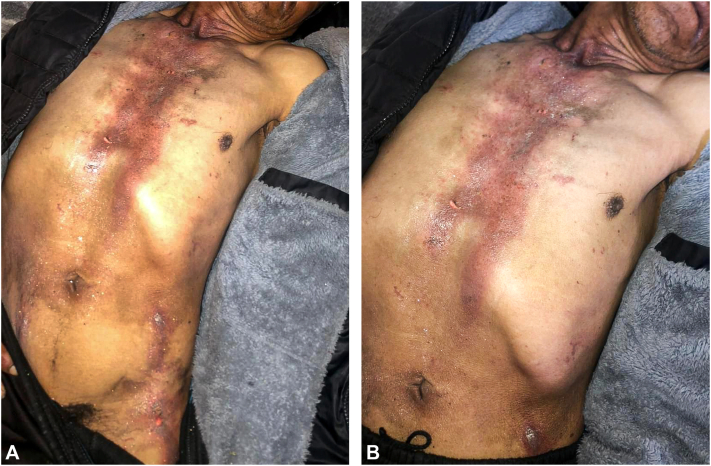
Lightning-induced keraunographic injury, acute presentation. **A,** Linear and curvilinear erythematous-violaceous lesions extending from the right supraclavicular region diagonally across the anterior chest toward the left lower abdomen, photographed 48 hours after lightning strike. **B,** Close view showing the superior origin of the keraunographic distribution at the right supraclavicular and neck junction.

**Fig 2:**
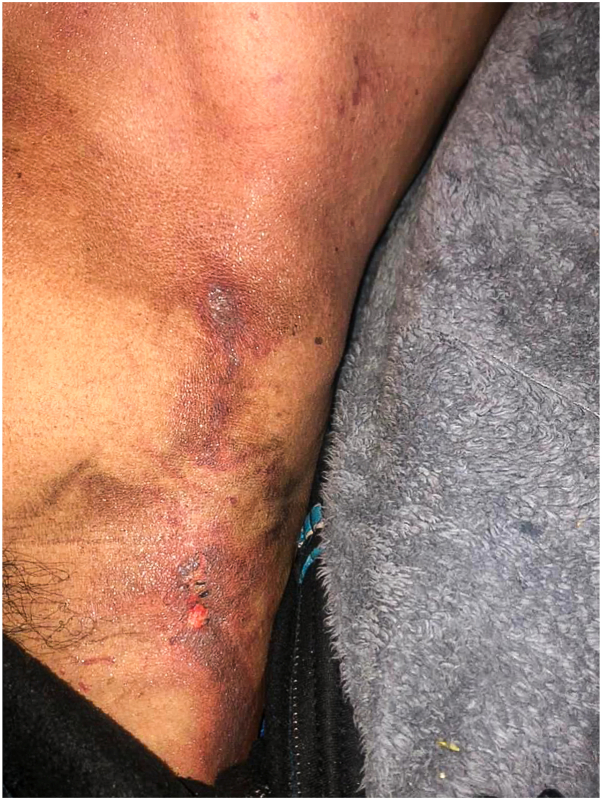
Lightning-induced keraunographic injury, acute presentation. Focal superficial erosions in the left inguinal region at the distal aspect of the cutaneous distribution.

**Fig 3:**
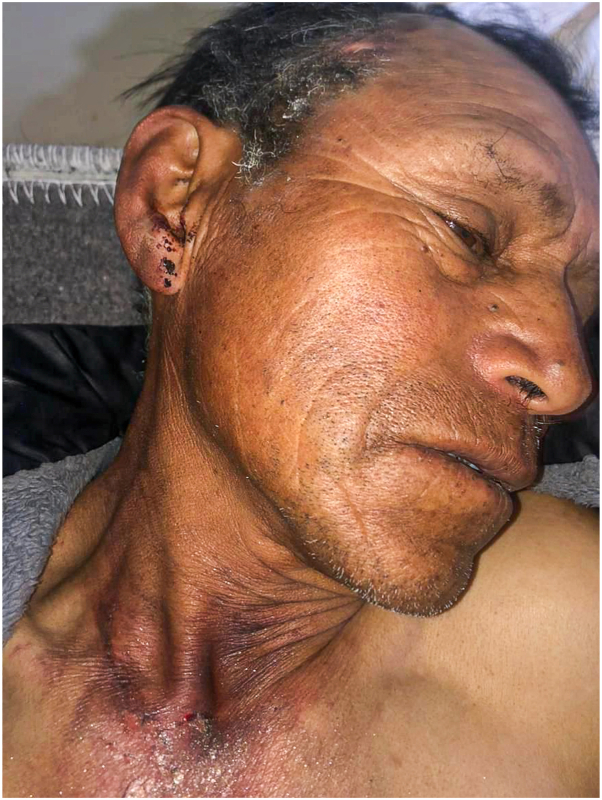
Lightning-induced otologic injury, acute presentation. Dried blood near the right external auditory canal at 48 hours, suggestive of barotraumatic otologic injury.

**Fig 4:**
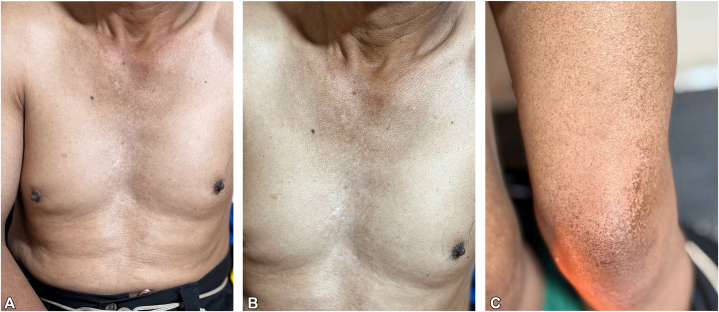
Lightning-induced keraunographic injury, 3-year follow-up. **A,** Anterior chest, wide view: subtle postinflammatory hypopigmentation along the midsternal keraunographic distribution without scarring or hypertrophic change, confirming resolution consistent with superficial flashover injury. **B,** Anterior chest, close-up: mixed postinflammatory dyspigmentation at the suprasternal origin of the distribution. **C,** Left lateral thigh: well-demarcated curvilinear postinflammatory hyper-hypopigmentation tracking the distal keraunographic distribution from the inguinal region, persisting without scarring more than 3 years after injury.

**Question: Which of the following best explains the absence of classic Lichtenberg figures in this patient?**

**A.** Absence of Lichtenberg figures 48 hours after injury makes lightning strike unlikely and favors a non-lightning thermal mechanism.
**B.** Lichtenberg figures represent full-thickness burns along superficial vessels and should persist as linear scars.
**C.** Lichtenberg figures are permanent and pathognomonic of a lightning strike; their absence excludes lightning as the diagnosis.
**D.** Lichtenberg figures are transient surface-discharge patterns that may dissipate before delayed evaluation; reduced chromatic contrast in darker phototypes may further limit their detection.
**E.** Keraunographic lesions and Lichtenberg figures share the same pathophysiological mechanism and always co-occur.

**Correct answer: D.** Lichtenberg figures are transient surface-discharge patterns that may dissipate before delayed evaluation; reduced chromatic contrast in darker phototypes may further limit their detection.

## Discussion

Keraunographic injury results from a flashover—a high-voltage discharge traveling across the body's external surface—producing linear, curvilinear, or branching erythematous-violaceous lesions without the deep thermal destruction of conducted current.^1^ Resolution without scarring, as documented in our patient, is consistent with the superficial nature of the flashover mechanism and distinguishes it from conducted electrical burns.

Lichtenberg figures—the fernlike arborescent patterns considered pathognomonic for lightning strike—represent a distinct variant of keraunographic findings.^2^ Histologic examination demonstrates normal epidermis and dermis with focal extravasation of red blood cells in the subcutaneous fat, supporting classification as a physical rather than thermal phenomenon.^3^ Importantly, Lichtenberg figures are highly evanescent, typically resolving within 48 hours of the strike, and their absence at clinical evaluation should not exclude lightning injury.^1^^,^^2^^,^^4^ In resource-limited settings without dermoscopy, lesion distribution, thunderstorm exposure, and associated findings anchor diagnosis.

In patients with darker skin (Fitzpatrick phototypes IV-VI), erythematous and violaceous changes exhibit reduced chromatic contrast, limiting early visual detection of Lichtenberg branching patterns and rendering residual dyspigmentation subtle at follow-up. These limitations highlight the necessity of images depicting skin of color in dermatology teaching materials to improve diagnostic familiarity.

This case also illustrates that lightning-related otologic injury may extend beyond tympanic membrane rupture and can be clinically associated with sensorineural hearing loss through acoustic trauma, barotrauma, and/or direct electrical effects.^4^^,^^5^ When audiometry is unavailable, tuning fork testing may help localize sensorineural hearing loss as a persistent sequela of lightning-related injury and support referral for otologic evaluation.

In summary, this case underscores that lightning injury should be recognized through a composite clinical pattern rather than a single classic sign.^5^ Classic Lichtenberg figures were not identified, yet the keraunographic distribution, otologic findings, and nonscarring resolution with persistent dyspigmentation supported a superficial flashover mechanism. Recognizing this pattern may help clinicians attribute delayed cutaneous and otologic findings to lightning injury even after pathognomonic surface-discharge patterns have resolved, particularly in darker phototypes and resource-limited settings.

## Conflicts of interest

None disclosed.
